# Effect of Linezolid on Clinical Severity and Pulmonary Cytokines in a Murine Model of Influenza A and *Staphylococcus aureus* Coinfection

**DOI:** 10.1371/journal.pone.0057483

**Published:** 2013-03-05

**Authors:** Xinyan Liu, Yaling He, Kun Xiao, Julie R. White, Dahlene N. Fusco, Genovefa A. Papanicolaou

**Affiliations:** 1 Infectious Disease Service, Memorial Sloan-Kettering Cancer Center, New York, New York, United States of America; 2 Laboratory of Comparative Pathology, Memorial Sloan-Kettering Cancer Center, New York, New York, United States of America; 3 Department of Medicine, Weill Cornell Medical College, Cornell University, New York, New York, United States of America; Charité, Campus Benjamin Franklin, Germany

## Abstract

Excessive inflammation contributes to the severity of post influenza pneumonia caused by methicillin resistant *S.aureus* (MRSA). Linezolid, vancomycin, and clindamycin are antibiotics used for MRSA infections. Linezolid has immunomodulatory properties. We report on the effects of the three antibiotics on microbial clearance, pulmonary cytokines and clinical course in a murine model of influenza and MRSA coinfection.

**Methods:**

B6 mice were infected with influenza A virus and 3 days later with MRSA, both intranasally. Treatment with placebo, linezolid, vancomycin or clindamycin started immediately after MRSA infection and continued for 72 hours. Bacterial and viral titers as well as cytokine concentrations in the lungs were assessed 4 and 24 hours after MRSA coinfection. Mice were weighted daily for 13 days.

**Results:**

Coinfected mice had increased pulmonary IL-1β, TNF-α and mKC at 4 and 24 hours, IL-6, IL-10 and IL-12 at 4 hours and IFN-γ at 24 hours after MRSA coinfection (all P<0.05). Compared to placebo, coinfected mice treated with linezolid, vancomycin or clindamycin had decreased pulmonary IL-6 and mKC at 4 hours and IFN-γ at 24 hours after MRSA coinfection (all P<0.05). IL-1β, TNF-α and IL-12 were similar in antibiotic-treated and placebo groups. All antibiotics similarly reduced MRSA without effect on influenza titers. Linezolid-treated mice had less weight loss on days 4–6 after influenza infection compared to placebo (all P<0.05). On all other days weight change was similar among all groups.

**Conclusions:**

This is the first report comparing the effects of antibiotics on cytokines and clinical outcome in a murine model of influenza and MRSA coinfection. Compared to placebo, antibiotic treatment reduced maximum concentration of IL-6, mKC and IFN-γ in the lungs without any difference among antibiotics. During treatment, only linezolid delayed weight loss compared to placebo.

## Introduction

Post-influenza *Staphylococcus aureus* pneumonia was identified as a common cause of death during the recent H1N1 influenza pandemic [1], [2]. Strains of community-acquired methicillin-resistant *Staphylococcus aureus* (CA-MRSA) have been associated with necrotizing pneumonia, particularly in children with influenza [3], [4]. Recent studies in murine models have emphasized the role of inflammation in lung histopathology and mortality during influenza and methicillin-resistant Staphylococcus aureus (MRSA) pneumonia [5], [6]. Lee et al. showed that influenza and MRSA coinfection lead to increased inflammation and more severe lung injury compared to MRSA alone. Such difference was most evident at 4 hours after MRSA infection [5]. In a different model, mice with post-influenza MRSA pneumonia had higher influenza titers compared to mice infected with influenza alone [6]. It is possible that immune dysregulation and subsequent ineffective but damaging inflammatory response play a prominent role in the pathogenesis of coinfection.

The innate immune system provides the first line of defense against *S. aureus* in the respiratory tract, a process mediated in part by cytokines [7]. Recent studies have furthered our understanding on the role of cytokines in regulating the inflammatory response to *S. aureus*. Ventura et al. reported increased concentrations of interleukin-1β (IL-1β), tissue necrosis factor-α (TNF-α), IL-6 and the mouse homologue of human IL-8 mouse keratinocyte chemoattractant (mKC), followed by recruitment of polymorphonuclear (PMN) cells to the lungs of mice evident by 30 minutes after intranasal challenge with *S. aureus*. By 6 hours, IL-1β, TNF-α, IL-6, and mKC were significantly increased. In contrast IL-10, IL-12p70 and interferon-γ (IFN-γ) were not significantly increased by 6 hours after inoculation [8]. IL-1β and TNF-α stimulate production of other cytokines. IL-1β is responsible for recruiting immune cells to the site of infection, and high concentrations of IL-1β have been reported to predispose to acute lung injury [9], [10]. Reduced levels of TNF-α, IL-1β and IL-6 have been associated with improved clearance of pulmonary staphylococci and survival [11]. IL-10 inhibits cytokine production by macrophages [12] and IFN-γ and IL-12 can promote phagocytic uptake and killing of *S. aureus* by immune dells [13]–[15].

Cytokines also play a key role in regulating the immune response to influenza and secondary bacterial infection [16]. IFN-γ is important for the development of helper T-cell type 1 (Th_1_) response. During influenza infection, IFN-γ contributes to macrophage dysfunction and ineffective killing of bacteria [17]. IFN-γ and IL-10 are involved in the regulation of anti-inflammatory response to influenza [18]–[20]. Increased susceptibility to secondary pneumococcal pneumonia has been, at least partially, ascribed to excessive IL-10 production and reduced neutrophil function in the lungs [21]. Use of macrolides, which are known to have immunomodulatory properties, has been associated with improved outcomes in post-influenza pneumococcal pneumonia [22]–[25]. Pharmacological interventions to regulate inflammation may have a role in the treatment of influenza and MRSA coinfection and warrant investigation.

At present a limited number of antibiotics are active against MRSA: Vancomycin, an inhibitor of cell wall synthesis, is considered first line therapy. Clindamycin, a protein synthesis inhibitor, is an oral alternative for CA-MRSA [26]. Linezolid is an oxazolidinone antibiotic with broad activity against Gram-positive organisms, including MRSA [27], [28]. Due to its excellent bioavailability, linezolid can be administered orally in the outpatient setting [29]. In a recent clinical trial comparing linezolid and vancomycin as treatment of primary MRSA pneumonia, linezolid was associated with superior microbiologic and clinical cure rates [30], [31]. The favorable outcomes observed with linezolid could be related to its immunomodulatory properties. *In vitro* linezolid inhibits lipopolysaccharide (LPS)- induced induction of proinflammatory cytokines in a concentration-dependent manner [32]. Treatment of periodontal disease with linezolid resulted in reduction in IL-1rα in the periapical dental tissue [33]. Potential differences among antibiotics on early induction of pro-inflammatory cytokines may be clinically important given the high mortality of influenza MRSA coinfection despite appropriate antibiotics [1], [2]. How antibiotic treatments influences early cytokine production and clinical outcome during influenza and MRSA coinfection has not been studied to date.

In the present study, we established a murine model of moderately-severe influenza MRSA coinfection and compared the effects of linezolid, vancomycin and clindamycin on bacterial titers, viral titers, pulmonary cytokines and clinical severity.

## Materials and Methods

### 2.1. Mice

Female C57BL6 mice were purchased from The Jackson Laboratory (MA, USA). All mice were 6–8 weeks old and weighed between 15–20 grams. Mice were housed in rooms with a 12∶12-hour light:dark cycle and were given free access to food and water. Animal care and experimental protocol were approved by the Institutional Animal Care and Use Committee of Memorial Sloan Kettering Cancer Center prior to initiation of experiments.

### 2.2. Pathogens

#### 2.2.1 Influenza

The Influenza A/WSN/33 (H1N1) strain of mouse-adapted influenza virus A was kindly provided by the laboratory of Dr. Peter Palese (Mt. Sinai Medical Center, New York, NY, USA). WSN was grown in Madin-Darby canine kidney (MDCK) cells as described [34]. A single stock of virus was used in this study. For determination of viral titers, both lungs were homogenized in 1 mL phosphate buffer saline (PBS) on ice and centrifuged at 4,000 rpm at 4°C for 15 minutes. Supernatants were frozen at −80°C. Viral titers were determined by a plaque forming assay performed in MDCK cells [35].

#### 2.2.2. Bacteria

The *S. aureus* strain ATCC-43300 (MRSA) was used for all experiments. ATCC-43300 is sensitive to linezolid and vancomycin but resistant to clindamycin. Bacteria were grown to the stationary phase at 37°C with constant shaking in tryptic soy broth. The resulting cultures were harvested by centrifugation, washed in PBS, resuspended in PBS with glycerol (10%), aliquoted to a final concentration 5×10^8^ colony-forming units (CFU)/mL, and stored at −80°C until use. For determination of bacterial titers in frozen stock or fresh tissues, samples were serial diluted, plated on mannitol salt agar (Sigma-Aldrich), and incubated at 37°C. CFU were determined at 48 h for bacterial colony counts. Viability of the inocula was confirmed by colony counts with each experiment.

### 2.3. Influenza and MRSA Coinfection Model

On Day 0 mice were anesthetized with 2.5% isoflurane and infected intranasally with 1,000 plaque-forming units (PFU) of WSN virus or phosphate buffered saline (PBS, 30 *µ*L/mouse). The infectious dose of 1,000 PFU WSN virus is non-lethal and causes weight loss of about 20% starting body weight [34]. Seventy-two hours (Day 3) after influenza infection, mice were anesthetized with 2.5% isoflurane and challenged intranasally with 9×10^6^ CFU MRSA or PBS (30 *µ*L/mouse).

Four infection groups were established: Flu+PBS (Influenza on Day 0 and PBS on Day 3); PBS+MRSA (PBS on Day 0 and MRSA on Day 3); Flu+MRSA (Influenza on Day 0 and MRSA on Day 3); and PBS (PBS on Day 0 and PBS on Day 3).

Body weight and general appearance were monitored daily for 13 days after influenza infection. Weights were measured every morning (between 9am to 11 am) for all experiments. In accordance with our institutional guidelines any animal with weight loss >25% starting body weight was sacrificed, and the infection was reported to be lethal [36].

### 2.4. Lung Histopathology

Lung histopathology was compared between Flu+PBS and Flu+MRSA mice. Mice were sacrificed 4 hours after MRSA coinfection. Lungs from each mouse were perfused with 10% buffered formalin, dissected into their respective lobes for maximum visualization, and histopathologic analysis was performed on all lobes. Formalin-fixed, paraffin-embedded 5-µm tissue sections were processed routinely and stained with hematoxylin and eosin (H&E). Lungs were evaluated by a veterinary pathologist blinded to the type of infection to determine the presence and degree of bronchiolar inflammation/necrosis, alveolar inflammation/necrosis, and perivascular inflammation.

### 2.5. Antibiotic Treatment

To assess the effect of antibiotics, mice were infected with influenza (Day 0) followed by MRSA infection 3 days later (Day 3) as described in 2.3. Antibiotics or placebo were administered subcutaneously starting immediately after MRSA infection. The following 4 treatment groups were compared: Lin: linezolid (Pfizer Inc, New York, NY, USA) 100 mg/kg every 12 hours; Van: vancomycin 180 mg/kg every 12 hours; Cli: clindamycin 300 mg/kg every 8 hours and Placebo: PBS as placebo given every 12 hours. All antibiotics and placebo were given subcutaneously. Antibiotics were administered for 72 hours.

### 2.6. Cytokine Analyses

Concentrations of IL-1β, IL-12p70, IFN-γ, IL-6, mKC, IL-10, and TNF-α in lung homogenates (prepared as described in 2.2.1.) and serum were measured by an ultra-sensitive mouse pro-inflammatory 7-plex kit from Meso Scale Discovery (MSD, Gaithersburg, MD, USA) according to the manufacturer’s instructions. Briefly, a spot on the base of each plate was pre-coated with a capture antibody for each cytokine. The standard and mouse samples (50 µl/well) were added to the prepared plates, and allowed to react at room temperature for 2 hours. Afterward, the plates were washed three times with washing buffer (1 × PBS with 0.05% Tween 20). Detection antibody was added and allowed to react at room temperature for 1 hour. After washing the plates three times and adding Read Buffer, the plates were analyzed on the MSD Sector Image 2400 (MSD). Calculation of cytokine concentrations was subsequently determined by 4-parameter logistic non-linear regression analysis of the standard curve.

### 2.7. Measurements and Statistics

All experiments were repeated at least two times with 5 mice per group. Mice were sacrificed at 4 and 24 hours after MRSA coinfection for measurement of bacterial titers, viral titers and cytokine concentrations. The percent body-weight change was calculated as the baseline weight minus the actual weight and divided by the baseline weight. CFU, PFU and cytokines concentrations were normalized by common logarithm transformation (log_10_). Zero values were offset by 0.1 to avoid undefined logarithm values.

We used two-tailed *Student’s t*-test for comparisons between two groups. One way analysis of variance (ANOVA) followed by post-hoc *t*-test with Bonferroni-adjustment were used for comparison among ≥3 groups. All statistical analyses were conducted with GraphPad Prism v5.1 (La Jolla, CA, USA). We consider P-value<0.05 as statistically significant.

## Results

### 3.1. Description of the Influenza and MRSA Coinfection Model

#### 3.1.1. Lung histopathology of mice with Flu+MRSA coinfection

**Figure 1:**
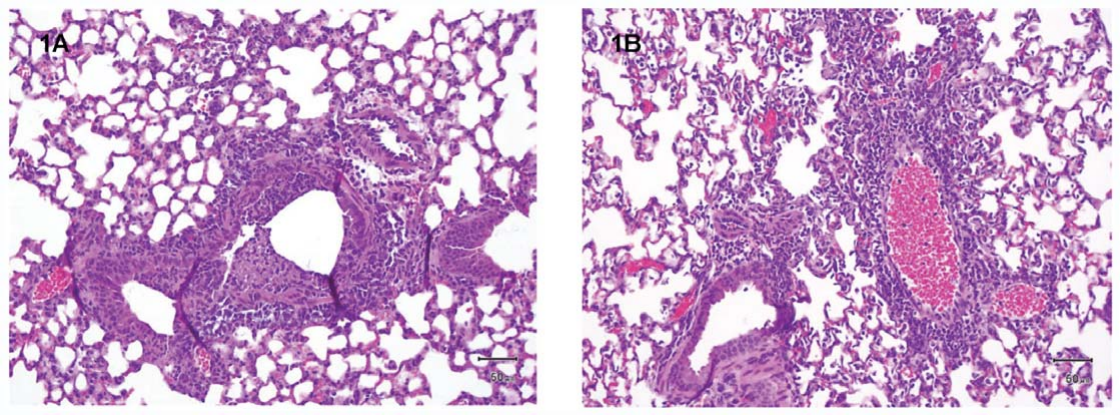
Lung histopathology of mice with Flu+MRSA coinfection, H&E. Mice were sacrificed at 4 hours after MRSA coinfection. Lungs were prepared as described in Materials and Methods 2.4, and evaluated by a veterinary pathologist blinded as to the type of infection. **A. Flu+PBS:** There is necrosis and sloughing of bronchiolar epithelial cells, with luminal accumulations of cellular debris and degenerate neutrophils. A peribronchiolar infiltrate of macrophages and neutrophils is present, along with mucinous degeneration of peribronchiolar connective tissue. **B. Flu+MRSA:** An infiltrate of neutrophils (many of which are degenerate) and macrophages primarily surrounds blood vessels and extends into the adjacent interstitium. Endothelial cells lining the blood vessels are often plump (reactive).

To confirm influenza and MRSA coinfection, we compared lung histopathology of Flu+PBS and Flu+MRSA mice on Day 3 after influenza infection and 4 hours after MRSA (or PBS) administration. Flu+PBS mice **(** **Figure 1A** **)** demonstrated typical bronchointerstitial pneumonia, with moderate bronchiolar epithelial cell necrosis and sloughing affecting approximately 30% of bronchioles, and luminal accumulation of nuclear and cellular debris, as well as neutrophils (many of which were degenerate). A mild to moderate multifocal infiltrate of macrophages with fewer neutrophils surrounded approximately 50% of bronchioles, with mucinous degeneration of peribronchiolar connective tissue. The inflammatory infiltrate extended into adjacent alveolar septa within more severely affected areas, with occasional septal necrosis and filling of alveoli with macrophages, degenerate neutrophils, and small amounts of fibrin. Perivascular inflammation was not a feature of Flu+PBS mice.

Flu+MRSA mice **(** **Figure 1B** **)** demonstrated a similar degree of bronchointerstitial pneumonia to the Flu+PBS mice. However, lungs from these mice also showed significant perivascular inflammation affecting approximately 15% of the lung. The inflammatory infiltrate, composed of neutrophils with fewer macrophages, widened perivascular connective tissue spaces and extended into the adjacent interstitium. Affected blood vessels were often lined by plump endothelial cells (reactive) and many vessels contained moderate numbers of neutrophils within their lumina.

#### 3.1.2. Weight change in mice with Flu+MRSA coinfection

**Figure 2:**
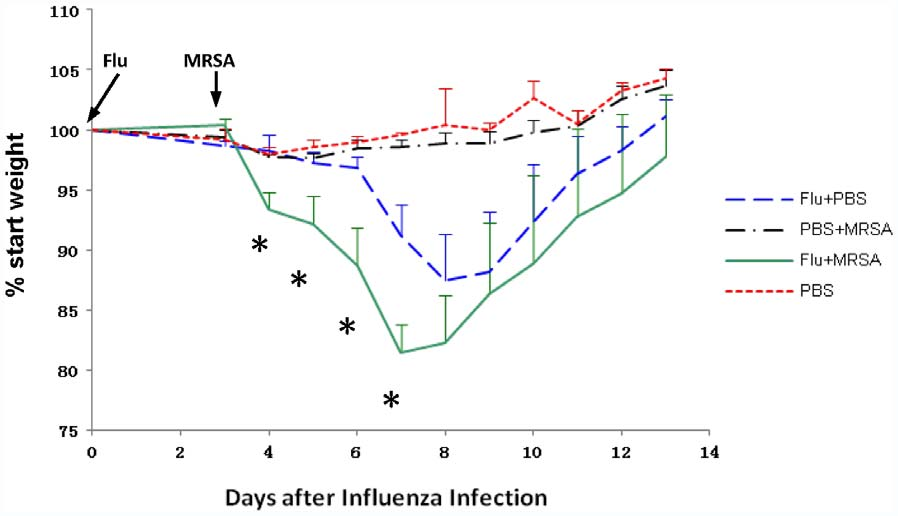
Weight change in mice with Flu+MRSA coinfection. Four groups of mice were infected with influenza (Flu) or PBS followed 3 days later by infection with MRSA or PBS as described in Materials and Methods, section 2.3. Mice were weighed daily between 9am and 11 am. Group average weight change is shown as mean+SEM (standard error of mean). Asterisks denote statistically significant difference between influenza alone (Flu+PBS) versus influenza and MRSA coinfected (Flu+MRSA) mice (P<0.05). The times of influenza and MRSA infection are indicated by arrows.

Flu+MRSA mice exhibited shivering and immobility by 6 hours after MRSA infection. Such symptoms resolved by 24 hours after MRSA infection. There was no mortality in any of the four infection groups. To assess the effect of coinfection on influenza-induced morbidity we compared weight loss over time between Flu+MRSA and Flu+PBS mice. Sham-infected (PBS) and MRSA-infected (PBS+MRSA) mice were included as controls (**Figure 2** **)**. PBS+MRSA and PBS mice had similar weight trends over time. By 7 days after influenza infection, Flu+PBS mice lost an average of 13% starting body weight compared to 18% for Flu+MRSA mice (P<0.05). Flu+MRSA mice had more weight loss on 4 to 7 days after influenza infection compared to Flu+PBS mice (all P<0.05).

#### 3.1.3. MRSA bacterial titer in the mice with Flu+MRSA coinfection

**Figure 3:**
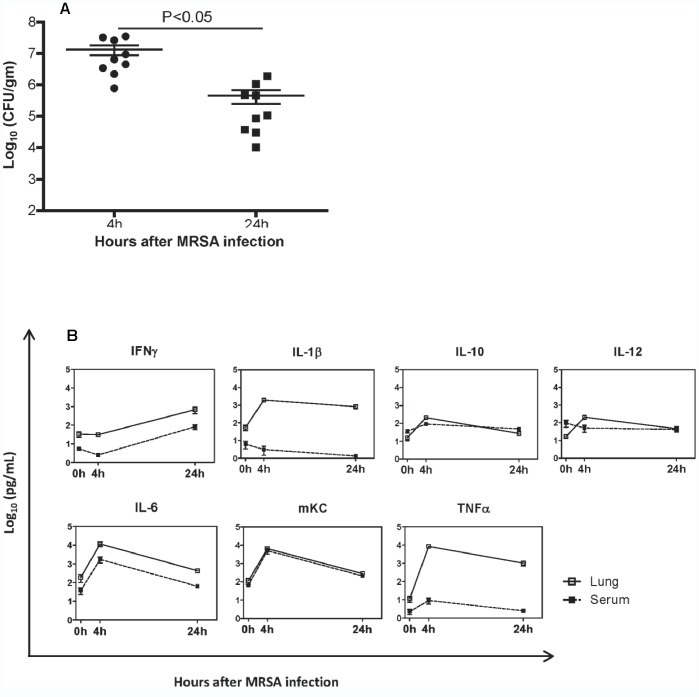
Flu+MRSA coinfected mice. Mice were infected with WSN influenza virus (Day 0) and 3 days later challenged intranasally with MRSA. Mice were sacrificed 4 or 24 hours after MRSA coinfection. **A.** **MRSA bacterial titers in mice with Flu+MRSA coinfection:** Each symbol represents one mouse. Horizontal bars represent first quartile, mean, and third quartile values. Y axis: log_10_ CFU per gram lung tissue; X axis: hours after MRSA infection. **B:** **Cytokine profile in the lungs and serum in mice with Flue+MRSA coinfection:** Cytokine concentrations in lung parenchyma (solid line) and serum (dotted line) were determined by a multiplex assay as described in Material and Methods. Squares represent mean cytokine concentration, Vertical bars: ± SEM. Y axis: log_10_ concentration (pg/ml), X axis: hours after MRSA infection.

Measurement of MRSA titers in the lungs of Flu+MRSA mice revealed a substantial decrease in CFU from 4 to 24 hours after MRSA infection **(** **Figure 3A, **P<0.001**)**.

#### 3.1.4. Cytokine profile in the lung and serum in mice with Flu+MRSA coinfection

The concentrations of lung and serum cytokines were determined in Flu+MRSA mice 3 days after influenza infection and prior to MRSA infection (0 hours), as well as 4 hours and 24 hours after MRSA coinfection. Results are shown in **Figure 3B** and **Table S1.**

Pulmonary concentrations of IL-1β, IL-10, IL-12, IL-6, mKC, and TNF-α were markedly elevated at 4 hours compared to concentrations at 0 hours (all P<0.0001). In contrast, the concentration of IFN-γ at 4 hours was similar to 0 hours (P = 0.98) but was significantly elevated at 24 hours after MRSA infection (P = 0.0066). The concentrations of pulmonary IL-1β, mKC, and TNF-α were lower at 24 hours compared to 4 hours (all P<0.05) but still higher than baseline (all P<0.05) **(** **Figure 3B** **)**.

Next we examined the effect of coinfection on serum cytokines. Serum concentrations of IL-10, IL-6 and mKC were markedly elevated at 4 hours compared to baseline (all p<0.05). At 24 hours after MRSA infection, serum mKC was still elevated (P = 0.001), whereas IL-10 and IL-6 returned to baseline (P = 0.13 and 0.12 respectively). Serum IFN-γ concentration was reduced at 4 hours compared to baseline (P = 0.007), but was markedly elevated at 24 hours, as compared to 4 hour and baseline (P = 0.0002 and 0.0028 respectively). Serum concentrations of IL-1β, IL-12 and TNF-α at 4 hours and 24 hours were similar to baseline.

Then we compare the cytokine concentrations in the lungs and serum. Concentrations of IFN-γ, IL-1β and TNF-α in the serum were lower than in lungs at baseline, and 4 and 24 hours after MRSA coinfection (**Table S2**, all P<0.05). In contrast the concentration of mKC was similar in serum and lungs at baseline, and 4 and 24 hours after MRSA coinfection. At baseline, we observed no difference between serum and pulmonary concentration for IL-6 and IL-10 (P = 0.1069 and 0.0527 respectively). At 4 and 24 hours after MRSA coinfection, however, both cytokines had higher pulmonary concentration than serum (both P<0.05). Pulmonary IL-12 was lower than serum at baseline (P = 0.0455), but it became higher than serum at 24 hours (P = 0.0057). We observed no difference in pulmonary and serum IL-12 levels at 24 hours after MRSA coinfection (P = 0.9797).

### 3.2. Effects of Antibiotic Treatment in Influenza and MRSA Coinfection

#### 3.2.1. MRSA and influenza titers in the lungs of Flu+MRSA coinfected mice treated with antibiotics

**Figure 4:**
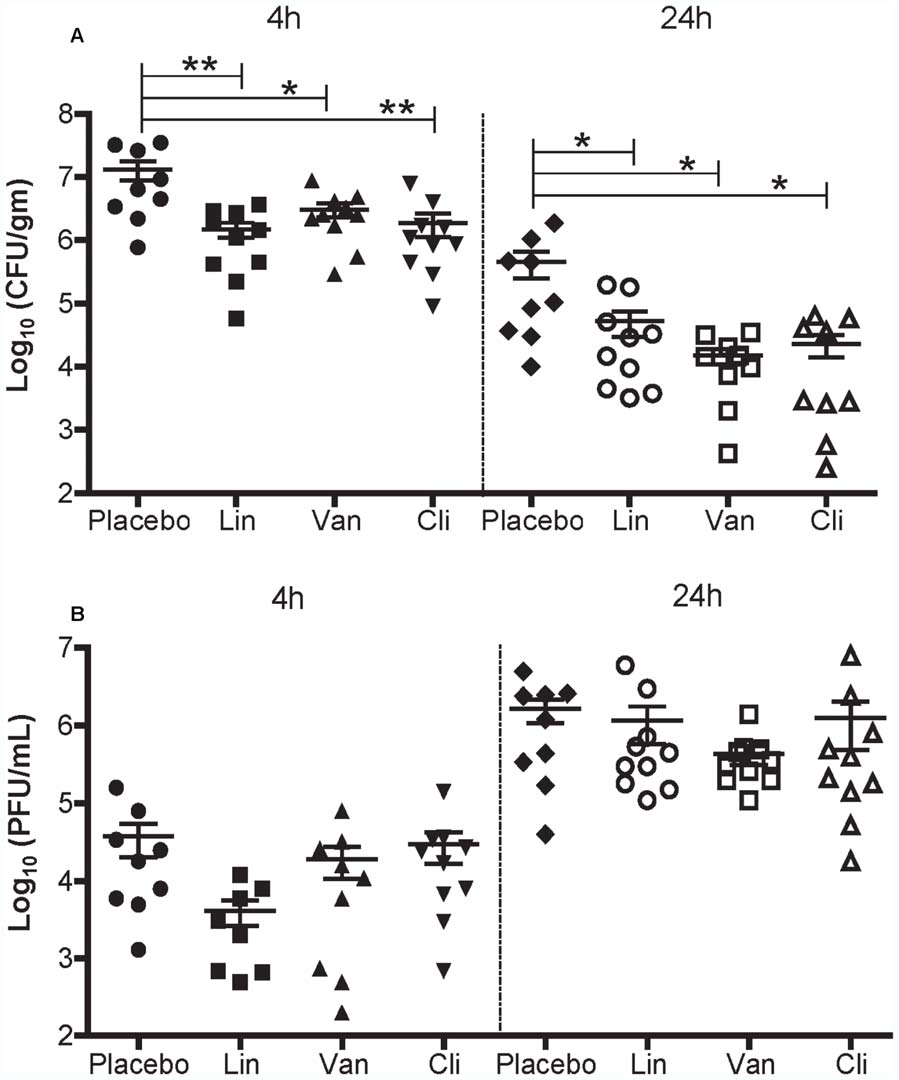
MRSA and influenza titers in the lungs of Flu+MRSA coinfected mice treated with antibiotics. Mice were infected with influenza (Day 0) and challenged with and 3 days later challenged intranasally with MRSA (Day 3). Treatment with placebo, linezolid (Lin), vancomycin (Van), or clindamycin (Cli) was started immediately after MRSA infection. Mice were sacrificed at 4 or 24 hours after MRSA coinfection. **A. MRSA bacterial titers** and **B. Influenza viral titers** in the lung at 4 hours and 24 hours after MRSA infection. Bacterial and viral titers were determined as indicated in Materials and Methods. Y axis: log_10_ (CFU/gm) for MRSA bacterial titers and log_10_ (PFU/mL) for influenza viral titers. X axis: Treatment groups. Asterisks indicate significant differences between groups (* P<0.05;** P<0.01).

We compared the effect of linezolid, vancomycin and clindamycin on MRSA bacterial titers in the lungs of Flu+MRSA mice **(** **Figure 4A** **)**. Antibiotic-treated mice had lower CFU of MRSA in the lungs compared to placebo at 4 hours (all P<0.05) and 24 hours (all P<0.05) after MRSA infection. There was no significant difference in the MRSA bacterial titers among the 3 antibiotics.

To assess whether antibiotic treatment had any indirect antiviral effect we compared influenza titers in the lungs among the four groups of mice **(** **Figure 4B** **)**. There was no statistically significant difference in influenza viral titers among the four treatment groups at 4 or 24 hours after MRSA infection (ANOVA P = 0.069 and 0.5185 respectively). At 4 hours after MRSA infection, linezolid treated mice had an approximately 1-log lower PFU compared to other treatment groups, albeit the difference is not statistically significant.

#### 3.2.2. Cytokine profile in the lungs of Flu+MRSA coinfected mice treated with antibiotics

**Figure 5:**
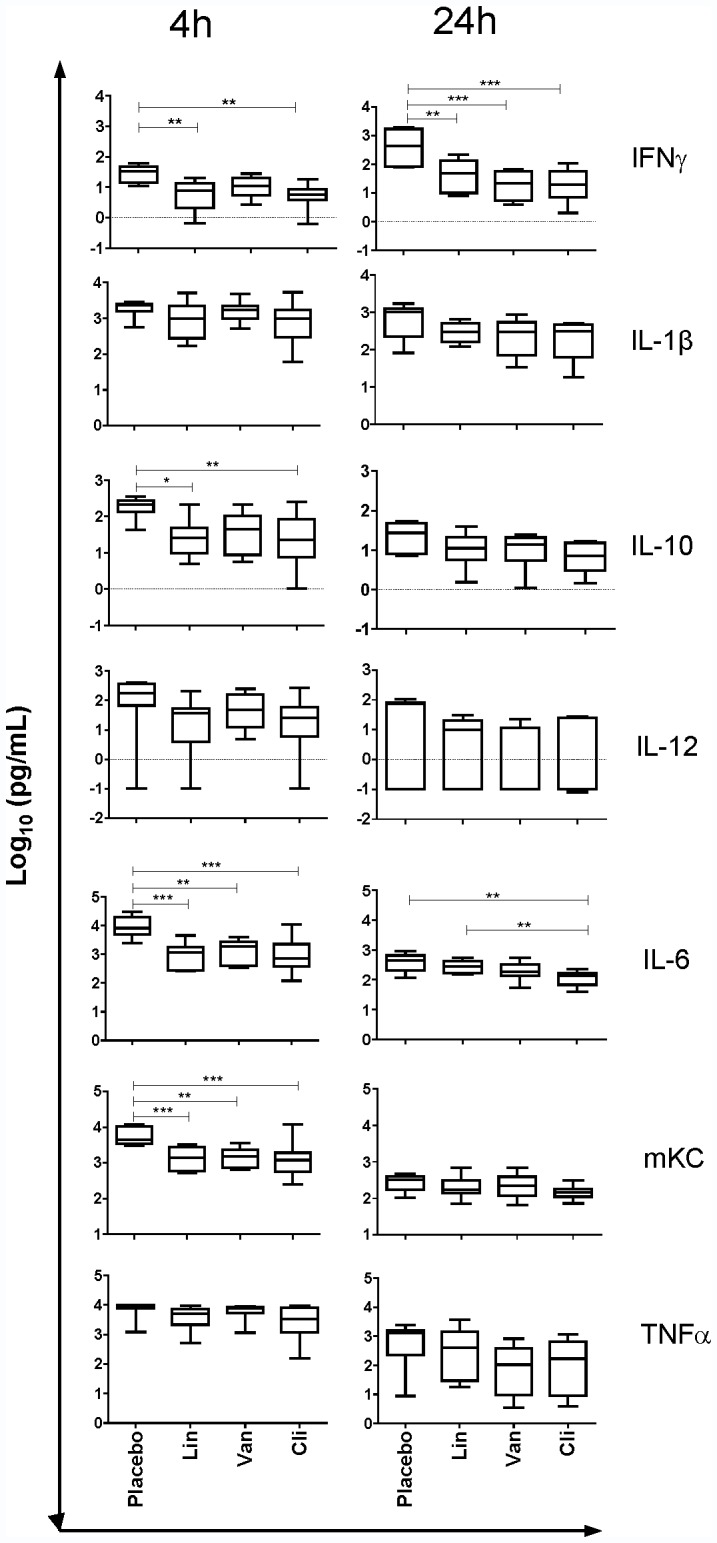
Cytokine profile in the lungs in Flu+MRSA coinfected mice treated with antibiotics. Influenza and MRSA coinfected mice (Flu+MRSA) were treated with placebo, linezolid (Lin), vancomycin (Van), or clindamycin (Cli) starting immediately after MRSA infection. Boxplots show cytokine concentrations in lungs at 4 or 24 hours after MRSA coinfection. Y axis: log_10_ cytokine concentration (pg/mL). X axis: Treatment groups. Asterisks indicate significant differences between groups (* P<0.05;** P<0.01; *** P<0.001).

We compared concentrations of pulmonary cytokines among the four treatment groups at 4 and 24 hours after MRSA infection **(** **Figure 5** **).** At 4 hours after MRSA coinfection, linezolid, vancomycin or clindamycin-treated mice had lower concentrations of IL-6 and mKC in the lungs compared to placebo (all P<0.01). Linezolid and clindamycin were associated with decreased concentrations of IL-10 and IFN-γ compared to placebo (all P<0.05). At 4 hours there was no significant difference in the concentrations of IL-1β, TNF-α and IL-12 among the four treatment groups (ANOVA P = 0.2450, 0.0768, and 0.2485 respectively).

At 24 hours after MRSA coinfection, linezolid, vancomycin or clindamycin-treated mice had lower concentrations of IFN-γ compared to placebo (all P<0.01). Clindamycin-treated mice had significantly lower IL-6 concentration as compared to linezolid- or placebo-treated mice (both p<0.01). There was no significant difference in the concentrations of IL-1β, TNF-α, mKC, IL-6 and IL-12 among the four groups (ANOVA P = 0.0999, 0.2249, 0.2478, 0.1024, and 0.2876 respectively).

#### 3.2.3. Weight change in Flu+MRSA coinfected mice treated with antibiotics

**Figure 6:**
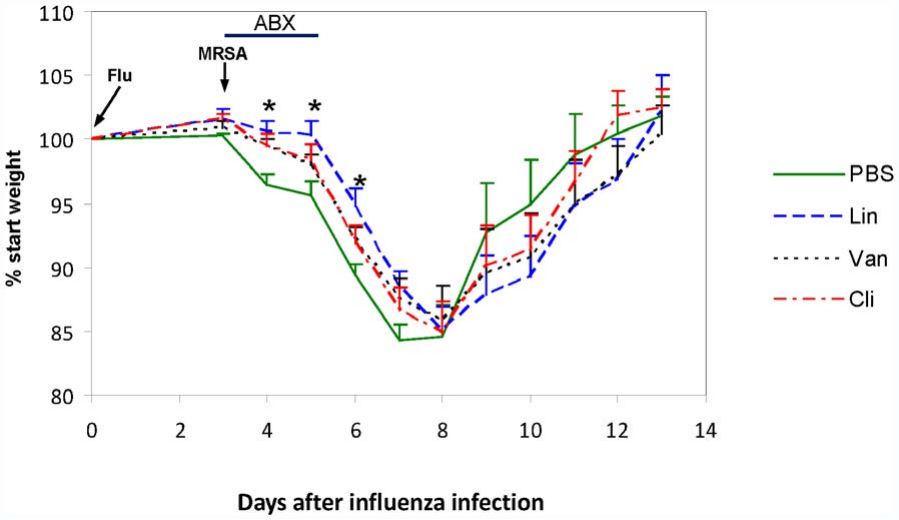
Weight change in Flu+MRSA coinfected mice treated with antibiotics. Influenza and MRSA coinfected (Flu+MRSA) mice were treated with placebo, linezolid (Lin), vancomycin (Van), or clindamycin (Cli) starting immediately after MRSA infection and continuing for 3 days. Mice were weighed daily between 9am and 11 am. Group average weight change is shown as mean+SEM. Asterisks denote significant difference between linezolid-treated and placebo treated mice (P<0.05). Times of influenza (Flu) and MRSA infection are indicated by arrows. Duration of antibiotic treatment (ABX) is indicated by horizontal line.

There was no mortality n any of the four treatment groups. **Figure 6** shows body weight changes over time for the four treatment groups. On Days 4 through 6, the days of antibiotic treatment, linezolid-treated mice lost less weight compared to placebo-treated mice (all P<0.05). In contrast, there was no significant difference in weight loss between vancomycin or clindamycin-treated mice, compared to placebo, during this time. There was no statistical difference among the four treatment groups at any other time point. In mice treated with placebo maximum weight loss was observed on Day 7, compared to Day 8 in mice that had been treated with linezolid, vancomycin or clindamycin. Eventually all groups had similar weight loss and recovered fully back to their baseline weight.

## Discussion

Bacterial pneumonia following influenza infection carries substantial morbidity and mortality [4]. The importance of *S. aureus* as a cause of post-influenza pneumonia has been re-emphasized since the 2009 H1N1 pandemic when *S. aureus* was a common co-existing pathogen in fatal cases [1]. Two recently described murine models of influenza and MRSA pneumonia suggest that an ineffective and damaging inflammatory response to infection contributes to lung injury [5], [6]. This suggests that immunomodulators may be of use in ameliorating excessive inflammation and improving clinical outcomes [22], [24], [25].

A limited number of antibiotics are active against MRSA. The Infectious Disease Society of America recommends vancomycin as first line treatment for severe MRSA infections, and clindamycin as an alternative for treating CA-MRSA [26]. Linezolid is an antibiotic approved by the FDA for the treatment of MRSA and known to have immunomodulatory properties [27]–[29], [32], [33]. Interestingly, a randomized trial showed that linezolid was superior to vancomycin for treating primary MRSA pneumonia [30]. Currently there is no data regarding the optimal antibiotic choice for post-influenza MRSA pneumonia. Antibiotics are often prescribed empirically during influenza prior to confirmation of bacterial coinfection, yet the impact of antibiotics on the clinical course of influenza is unclear.

In the present study we established a murine model of influenza and MRSA coinfection and compared the effects of vancomycin, linezolid, and clindamycin on bacterial and viral titers, pulmonary cytokines and clinical course. A regimen of linezolid 100–120 mg/kg twice daily produces epithelial lining fluid exposures comparable to human exposures following regimens of linezolid 600 mg twice daily [37]. We studied a moderately severe coinfection as the majority of patients who receive antibiotic treatment do not have fatal infection.

Examination of lungs of coinfected mice confirmed histopathologic features consistent with coinfection. Examination of the lungs of influenza infected (Flu+PBS) mice demonstrated typical lesions of influenza infection, composed of necrotizing bronchointerstitial pneumonia, which was primarily centered on bronchioles and extended into adjacent alveoli. In contrast Flu+MRSA mice showed necrotizing bronchointerstitial pneumonia and an inflammatory process surrounding blood vessels, which was also present in areas of the lung that were unaffected by bronchointerstitial pneumonia. A perivascular pattern of inflammation is consistent with the hematogenous spread of a bacterial pathogen such as MRSA, rather than being due to influenza alone.

Among the parameters available for evaluation of influenza severity in mice, body weight loss and mortality are most commonly used [38]. Influenza infection leading to a 25% weight loss is considered fatal [36]. In our model influenza and MRSA coinfection was associated with greater weight loss compared to influenza alone. In contrast, MRSA infection alone was not associated with weight loss. Thus, the increased weight loss observed in Flu+MRSA (versus Flu+PBS) is likely reflective of increased influenza severity, rather than a clinical response to bacterial infection. This finding underscores the importance of examining not only bacterial but also ongoing viral pathology in the setting of post-influenza bacterial pneumonia.

Interestingly viral titers in Flu+MRSA coinfected mice on day 3 and 4 after influenza infection were approximately 10 fold higher than predicted with influenza infection alone using the same dose of influenza in our own laboratory [34]. This data further supports our observation that MRSA coinfection potentiated the severity of influenza. In agreement with published literature, MRSA infection alone was not associated with sustained morbidity in our model [39].

MRSA titers in the lungs of Flu+MRSA coinfected mice 4 hours after MRSA coinfection were comparable to the titers reported in B6 mice after intranasal challenge with a different strain of *S. aureus* and a 3-fold higher inoculum [8]. Antecedent influenza infection possibly contributed to increased susceptibility to MRSA infection in our model. Resistance to *S. aureus* in B6 mice is dependent on neutrophils [40] and influenza infection is known to impair neutrophil function [41]. Impaired mucosal immunity may have further facilitated pulmonary infection with MRSA [42].

Next we characterized the early pulmonary cytokine profile in Flu+MRSA coinfected mice. The concentrations of pulmonary IL-1β, IL-6, mKC, and TNF-α were significantly increased at 4 hours after MRSA coinfection. In contrast, compared to baseline, pulmonary IFNy was not increased at 4 hour after MRSA infection, but was elevated by 24 hours. We noticed an increase in the concentrations of anti-inflammatory IL-10 and IL-12p70 at 4 hours. However their concentration returned to baseline at 24 hours after MRSA infection.

After validating our coinfection model, we compared the effects of linezolid, vancomycin, clindamycin and placebo in bacterial and viral clearance, cytokine secretion and clinical outcome. We show that antibiotic treatment reduced pulmonary MRSA titers in coinfected mice without significant differences among the three antibiotics. None of the antibiotics had an appreciable effect on influenza titers in the lungs compared to placebo.

A main objective of our study was to compare the impact of antibiotics on early cytokines during influenza and MRSA coinfection. IL-1β and TNF- α are early cytokines that regulate neutrophil recruitment and downstream cytokines [8]–[11], [43]. IL-12 interferes with phagocytic uptake of *S. aureus* [13]–[15]. In our study, antibiotic treatment did not have any impact on pulmonary IL-1β, TNF-α or IL-12. However, antibiotic treatment reduced the maximal levels of pulmonary IL-6, mKC, and IFNy. These three cytokines are associated with recruitment of neutrophils to the lungs [44]. In a model of influenza and MRSA coinfection, higher concentration of IFNy correlated with greater pathologic damage [5], therefore lowering IFNy may be beneficial. However, IFN-γ also plays a an antiviral role against influenza, including a major role in the development of anti-influenza Th_1_ response [45]. Further studies with a model of severe coinfection could assess the net effect of antibiotic treatment on antiviral response, including cellular recruitment to the lungs.

In our coinfection model, IL-10 was only increased at 4 hours and back to baseline at 24 hours after coinfection. Compared to placebo, linezolid and clindamycin, but not vancomycin, were associated with decreased concentration of IL-10 at 4 hours after MRSA infection. During acute influenza infection IL-10 is produced in the lungs by influenza-specific T-cells. Recent studies have shown a potential role for IL-10 inhibition in decreased immune mediated pathology and better clinical outcomes of influenza [20]. Thus inhibition of IL-10 by linezolid and clindamycin could have clinical implications later in the course of coinfection. Additional studies are needed to confirm a potential role of linezolid or clindamycin in the regulation of IL-10.

Lastly we examined the effect of antibiotics on the clinical course of coinfection. We showed that antibiotic- and placebo-treated mice had similar trends in weight change over time. The placebo treatment group reached maximal weight loss on Day 7, whereas antibiotic treatment groups had maximal weight loss on Day 8. Treatment with linezolid was associated with a mild but statistically significant delay in weight loss compared to placebo. Specifically linezolid-treated mice lost less weight on days 4–6 after influenza infection, yet eventually they lost a similar amount of weight compared to the other three groups. Interestingly the delay in weight loss was noted only during the days of exposure to linezolid. Further studies are needed to assess if treatment with linezolid beyond 72 hours would be associated with sustained difference in weight loss beyond Day 6. Although antibiotic-treated mice appeared to have a delay in regaining weight compared to placebo, the difference was not statistically significant. All treatment groups had regained weights similarly on Day 13.

To our knowledge, this is the first report comparing the effects of three antibiotics commonly used clinically for post influenza MRSA pneumonia on microbiologic, clinical and immunological parameters of coinfection. In our model we did not appreciate any differential impact among antibiotics on bacterial or viral titers or maximal weight loss. We show that all three antibiotics decreased the maximal levels of pulmonary IL-6, mKC and IFN-γ, which play key roles in host immune response to coinfection.

Our study has several limitations. First, antibiotics were given for a total of 72 hours starting at the time of MRSA coinfection. It is possible that prolonging antibiotic treatments beyond 72 hours could influence cytokines that increase later in the course of influenza, such as IL-10. It is also possible that the transient delay in weight loss could be sustained with prolonged antibiotic treatment, resulting in less maximum weight loss. Future studies with longer treatment duration and assessment of cytokines at later time points could provide such information. Second, examination of additional parameters such as oxygen saturation and histopathology scores could provide further insights on the effect of antibiotics on severity of coinfection [38], [46]. Our model of moderately severe coinfection is clinically relevant because the majority of coinfections in humans are non-lethal. Further studies employing a model of lethal coinfection could assess the impact of antibiotics on survival. Third, we only tested a single regimen of linezolid to simulate clinical exposures [37]. Different regimen of linezolid may enhance potential immunomodulatory effects.

In summary, we show that a short course of linezolid, vancomycin and clindamycin had similar effects on bacterial clearance and overall weight loss in a murine model of moderately severe influenza and MRSA coinfection. Compared to placebo, antibiotic treatment reduced maximum concentration of IL-6, mKC and IFN-γ in the lungs without any difference among the antibiotics. During treatment, only linezolid delayed weight loss as compared to placebo. Because the frequency of MRSA as a co-pathogen in post influenza pneumonia is increasing, further studies are warranted to optimize antibiotic treatment and outcomes.

## Supporting Information

Table S1 **Lung and serum cytokine concentrations were measured by a multiplex ELISA at 0 hours (3 days after influenza influenza), 4 hours and 24 hours after MRSA challenge.** Numbers: cytokine concentration Mean ± SEM (pg/mL). Concentrations of each cytokine between time-points are compared by unpaired Student’s t-test on logarithmic data. P values <0.05 are considered significant and reported.
(PDF)

Table S2 **Lung and serum cytokine concentrations were measured by a multiplex ELISA at 0 hours (3 days after influenza influenza), 4 hours and 24 hours after MRSA infection.** Numbers: cytokine concentration Mean ± SEM (pg/mL). Concentrations of each cytokine in the lungs and serum are compared by unpaired Student’s t-test on logarithmic data. P values <0.05 are considered significant and reported.
(PDF)

## References

[1] Murray RJ, Robinson JO, White JN, Hughes F, Coombs GW, et al (2010) Community-acquired pneumonia due to pandemic A(H1N1)2009 influenzavirus and methicillin resistant Staphylococcus aureus co-infection. PLoS One 5: e8705. 20090931 10.1371/journal.pone.0008705 PMC2806836

[2] Centers for Disease Control and Prevention (CDC) (2009) Bacterial coinfections in lung tissue specimens from fatal cases of 2009 pandemic influenza A (H1N1)–United States, May-August 2009. MMWR Morb Mortal Wkly Rep 58: 1071–1074. 19798021

[3] Finelli L, Fiore A, Dhara R, Brammer L, Shay DK, et al (2008) Influenza-associated pediatric mortality in the United States: increase of Staphylococcus aureus coinfection. Pediatrics 122: 805–811. 18829805 10.1542/peds.2008-1336

[4] Centers for Disease Control and Prevention (CDC) (2007) Severe methicillin resistant Staphylococcus aureus community-acquired pneumonia associated with influenza-Louisiana and Georgia, December 2006-January 2007. MMWR Morb Mortal Wkly Rep 56: 325–329. 17431376

[5] Lee MH, Arrecubieta C, Martin FJ, Prince A, Borczuk AC, et al (2010) A postinfluenza model of Staphylococcus aureus pneumonia. J Infect Dis 201: 508–515. 20078212 10.1086/650204 PMC3664424

[6] Iverson AR, Boyd KL, McAuley JL, Plano LR, Hart ME, et al (2011) Influenza virus primes mice for pneumonia from Staphylococcus aureus. J Infect Dis 203: 880–888. 21278211 10.1093/infdis/jiq113 PMC3071123

[7] Yao L, Berman JW, Factor SM, Lowy FD (1997) Correlation of histopathologic and bacteriologic changes with cytokine expression in an experimental murine model of bacteremic Staphylococcus aureus infection. Infect Immun 65: 3889–3895. 9284168 10.1128/iai.65.9.3889-3895.1997 PMC175555

[8] Ventura CL, Higdon R, Hohmann L, Martin D, Kolker E, et al (2008) Staphylococcus aureus elicits marked alterations in the airway proteome during early pneumonia. Infect Immun 76: 5862–5872. 18852243 10.1128/IAI.00865-08 PMC2583584

[9] Bubeck Wardenburg J, Schneewind O (2008) Vaccine protection against Staphylococcus aureus pneumonia. J Exp Med 205: 287–294. 18268041 10.1084/jem.20072208 PMC2271014

[10] Goodman RB, Pugin J, Lee JS, Matthay MA (2003) Cytokine-mediated inflammation in acute lung injury. Cytokine Growth Factor Rev 14: 523–535. 14563354 10.1016/s1359-6101(03)00059-5

[11] Banerjee A, Stevenaert F, Pande K, Haghjoo E, Antonenko S, et al (2010) Modulation of paired immunoglobulin-like type 2 receptor signaling alters the host response to Staphylococcus aureus-induced pneumonia. Infect Immun 78: 1353–1363. 20065029 10.1128/IAI.00969-09 PMC2825905

[12] Fiorentino DF, Zlotnik A, Mosmann TR, Howard M, O’Garra A (1991) IL-10 inhibits cytokine production by activated macrophages. J Immunol 147: 3815–3822. 1940369

[13] Aoki N, Xing Z (2004) Use of cytokines in infection. Expert Opin Emerg Drugs 9: 223–236. 15571481 10.1517/14728214.9.2.223

[14] Zhao YX, Nilsson IM, Tarkowski A (1998) The dual role of interferon-gamma in experimental Staphylococcus aureus septicaemia versus arthritis. Immunology 93: 80–85. 9536122 10.1046/j.1365-2567.1998.00407.x PMC1364109

[15] Yamada M, Gomez JC, Chugh PE, Lowell CA, Dinauer MC, et al (2011) Interferon-gamma production by neutrophils during bacterial pneumonia in mice. Am J Respir Crit Care Med 183: 1391–1401. 21169470 10.1164/rccm.201004-0592OC PMC3114063

[16] Parker D, Prince A (2011) Innate immunity in the respiratory epithelium. Am J Respir Cell Mol Biol 45: 189–201. 21330463 10.1165/rcmb.2011-0011RT PMC3175551

[17] van der Sluijs KF, van der Poll T, Lutter R, Juffermans NP, Schultz MJ (2010) Bench-to-bedside review: bacterial pneumonia with influenza - pathogenesis and clinical implications. Crit Care 14: 219. 20459593 10.1186/cc8893 PMC2887122

[18] Hennet T, Ziltener HJ, Frei K, Peterhans E (1992) A kinetic study of immune mediators in the lungs of mice infected with influenza A virus. J Immunol 149: 932–939. 1321855

[19] Fritz RS, Hayden FG, Calfee DP, Cass LM, Peng AW, et al (1999) Nasal cytokine and chemokine responses in experimental influenza A virus infection: results of a placebo-controlled trial of intravenous zanamivir treatment. J Infect Dis 180: 586–593. 10438343 10.1086/314938

[20] Sun J, Madan R, Karp CL, Braciale TJ (2009) Effector T cells control lung inflammation during acute influenza virus infection by producing IL-10. Nat Med 15: 277–284. 19234462 10.1038/nm.1929 PMC2693210

[21] van der Sluijs KF, van Elden LJ, Nijhuis M, Schuurman R, Pater JM, et al (2004) IL-10 is an important mediator of the enhanced susceptibility to pneumococcal pneumonia after influenza infection. J Immunol 172: 7603–7609. 15187140 10.4049/jimmunol.172.12.7603

[22] Mufson MA, Stanek RJ (2006) Revisiting combination antibiotic therapy for community-acquired invasive Streptococcus pneumoniae pneumonia. Clin Infect Dis 42: 304–306. 16355349 10.1086/499110

[23] Martinez FJ (2004) Monotherapy versus dual therapy for community-acquired pneumonia in hospitalized patients. Clin Infect Dis 38 Suppl 4S328–340. 15127366 10.1086/382689

[24] Baddour LM, Yu VL, Klugman KP, Feldman C, Ortqvist A, et al (2004) Combination antibiotic therapy lowers mortality among severely ill patients with pneumococcal bacteremia. Am J Respir Crit Care Med 170: 440–444. 15184200 10.1164/rccm.200311-1578OC

[25] Karlstrom A, Boyd KL, English BK, McCullers JA (2009) Treatment with protein synthesis inhibitors improves outcomes of secondary bacterial pneumonia after influenza. J Infect Dis 199: 311–319. 19113989 10.1086/596051 PMC2687083

[26] Liu C, Bayer A, Cosgrove SE, Daum RS, Fridkin SK, et al (2011) Clinical practice guidelines by the infectious diseases society of america for the treatment of methicillin-resistant Staphylococcus aureus infections in adults and children: executive summary. Clin Infect Dis 52: 285–292. 21217178 10.1093/cid/cir034

[27] Barman TK, Pandya M, Mathur T, Bhadauriya T, Rao M, et al (2009) Novel biaryl oxazolidinones: in vitro and in vivo activities with pharmacokinetics in an animal model. Int J Antimicrob Agents 33: 280–284. 19091517 10.1016/j.ijantimicag.2008.08.025

[28] Leach KL, Brickner SJ, Noe MC, Miller PF (2011) Linezolid, the first oxazolidinone antibacterial agent. Ann N Y Acad Sci 1222: 49–54. 21434942 10.1111/j.1749-6632.2011.05962.x

[29] Welshman IR, Sisson TA, Jungbluth GL, Stalker DJ, Hopkins NK (2001) Linezolid absolute bioavailability and the effect of food on oral bioavailability. Biopharm Drug Dispos 22: 91–97. 11745911 10.1002/bdd.255

[30] Wunderink RG, Niederman MS, Kollef MH, Shorr AF, Kunkel MJ, et al (2012) Linezolid in methicillin-resistant Staphylococcus aureus nosocomial pneumonia: a randomized, controlled study. Clin Infect Dis 54: 621–629. 22247123 10.1093/cid/cir895

[31] Wunderink RG, Rello J, Cammarata SK, Croos-Dabrera RV, Kollef MH (2003) Linezolid vs vancomycin: analysis of two double-blind studies of patients with methicillin-resistant Staphylococcus aureus nosocomial pneumonia. Chest 124: 1789–1797. 14605050

[32] Garcia-Roca P, Mancilla-Ramirez J, Santos-Segura A, Fernandez-Aviles M, Calderon-Jaimes E (2006) Linezolid diminishes inflammatory cytokine production from human peripheral blood mononuclear cells. Arch Med Res 37: 31–35. 16314183 10.1016/j.arcmed.2005.05.022

[33] Danin J, Linder L, Lundqvist G, Wretlind B (2003) Cytokines in periradicular lesions: the effect of linezolid treatment. Oral Surg Oral Med Oral Pathol Oral Radiol Endod 96: 492–498. 14561977 10.1016/s1079-2104(03)00059-3

[34] Fusco D, Liu X, Savage C, Taur Y, Xiao W, et al (2010) Echinacea purpurea aerial extract alters course of influenza infection in mice. Vaccine 28: 3956–3962. 20382242 10.1016/j.vaccine.2010.03.047 PMC3016056

[35] Tobita K, Sugiura A, Enomote C, Furuyama M (1975) Plaque assay and primary isolation of influenza A viruses in an established line of canine kidney cells (MDCK) in the presence of trypsin. Med Microbiol Immunol 162: 9–14. 1214709 10.1007/BF02123572

[36] Nguyen JT, Smee DF, Barnard DL, Julander JG, Gross M, et al (2012) Efficacy of combined therapy with amantadine, oseltamivir, and ribavirin in vivo against susceptible and amantadine-resistant influenza A viruses. PLoS One 7: e31006. 22292088 10.1371/journal.pone.0031006 PMC3264642

[37] Tessier PR, Keel RA, Hagihara M, Crandon JL, Nicolau DP (2012) Comparative in vivo efficacies of epithelial lining fluid exposures of tedizolid, linezolid, and vancomycin for methicillin-resistant Staphylococcus aureus in a mouse pneumonia model. Antimicrob Agents Chemother 56: 2342–2346. 22354302 10.1128/AAC.06427-11 PMC3346598

[38] Bouvier NM, Lowen AC (2010) Animal Models for Influenza Virus Pathogenesis and Transmission. Viruses 2: 1530–1563. 21442033 10.3390/v20801530 PMC3063653

[39] Skerrett SJ, Liggitt HD, Hajjar AM, Wilson CB (2004) Cutting edge: myeloid differentiation factor 88 is essential for pulmonary host defense against Pseudomonas aeruginosa but not Staphylococcus aureus. J Immunol 172: 3377–3381. 15004134 10.4049/jimmunol.172.6.3377

[40] von Kockritz-Blickwede M, Rohde M, Oehmcke S, Miller LS, Cheung AL, et al (2008) Immunological mechanisms underlying the genetic predisposition to severe Staphylococcus aureus infection in the mouse model. Am J Pathol 173: 1657–1668. 18974303 10.2353/ajpath.2008.080337 PMC2626378

[41] Sun K, Metzger DW (2008) Inhibition of pulmonary antibacterial defense by interferon-gamma during recovery from influenza infection. Nat Med 14: 558–564. 18438414 10.1038/nm1765

[42] Lijek RS, Weiser JN (2012) Co-infection subverts mucosal immunity in the upper respiratory tract. Curr Opin Immunol 24: 417–423. 22658762 10.1016/j.coi.2012.05.005 PMC3423578

[43] Marks JD, Marks CB, Luce JM, Montgomery AB, Turner J, et al (1990) Plasma tumor necrosis factor in patients with septic shock. Mortality rate, incidence of adult respiratory distress syndrome, and effects of methylprednisolone administration. Am Rev Respir Dis 141: 94–97. 2297191 10.1164/ajrccm/141.1.94

[44] Nathan C (2006) Neutrophils and immunity: challenges and opportunities. Nat Rev Immunol 6: 173–182. 16498448 10.1038/nri1785

[45] Schroder K, Hertzog PJ, Ravasi T, Hume DA (2004) Interferon-gamma: an overview of signals, mechanisms and functions. J Leukoc Biol 75: 163–189. 14525967 10.1189/jlb.0603252

[46] Verhoeven D, Teijaro JR, Farber DL (2009) Pulse-oximetry accurately predicts lung pathology and the immune response during influenza infection. Virology 390: 151–156. 19493556 10.1016/j.virol.2009.05.004 PMC2776688

