# Supplementary material for: Effect of Linezolid on Clinical Severity and Pulmonary Cytokines in a Murine Model of Influenza A and Staphylococcus aureus Coinfection
Source: PLoS One. 2013 Mar 5;8(3):e57483. doi: 10.1371/journal.pone.0057483 (PMC3589409; doi:10.1371/journal.pone.0057483)
Supplement: Table S1 — Lung and serum cytokine concentrations were measured by a multiplex ELISA at 0 hours (3 days after influenza influenza), 4 hours and 24 hours after MRSA challenge. Numbers: cytokine concentration Mean ± SEM (pg/mL). Concentrations of each cytokine between time-points are compared by unpaired Student’s t-test on logarithmic data. P values <0.05 are considered significant and reported. (PDF) [file pone.0057483.s001.pdf]

Supplement Table S1. Concentrations of cytokines in the lungs and serum of mice with influenza and MRSA coinfection

Lungs

Cytokine (pg/mL)	0h	4h	24h	P 0h vs 4h	P 4h vs 24h	P 0h vs 24h
**IFN-γ**	33.06±10.85	31.55±5.84	692.65±269.60	NS	0.0001	0.0066
**IL-1β**	56.60±19.62	1975.53±221.42	837.89±202.01	< 0.0001	0.0109	0.0016
**IL-10**	15.55±4.65	210.12±32.01	27.41±6.45	< 0.0001	< 0.0001	NS
**IL-12**	17.00±3.29	206.53±49.07	46.16±15.55	< 0.0001	0.0126	NS
**IL-6**	186.04±83.35	11767.72±3218.77	432.85±94.80	< 0.0001	< 0.0001	NS
**mKC**	116.07±40.42	6594.50±1189.87	286.09±45.40	< 0.0001	< 0.0001	0.0244
**TNFα**	11.37±4.21	8388.89±979.42	1010.11±302.49	< 0.0001	0.0008	0.0021

Serum

Cytokine (pg/mL)	0h	4h	24h	P 0h vs 4h	P 4h vs 24h	P 0h vs 24h
**IFN-γ**	5.42±0.76	2.53±0.34	83.16±24.84	0.0070	0.0002	0.0028
**IL-1β**	6.34±2.95	3.13±1.62	1.35±0.13	NS	NS	NS
**IL-10**	36.69±7.51	91.42±18.18	49.28±3.37	0.0212	0.0377	NS
**IL-12**	95.81±40.49	50.31±21.35	41.94±5.61	NS	NS	NS
**IL-6**	37.59±14.33	1735.74±614.42	64.15±9.26	0.0005	0.0001	NS
**mKC**	64.08±11.89	5058.35±1793.54	210.73±25.40	< 0.0001	0.0002	0.0010
**TNFα**	2.26±0.75	8.97±3.28	2.51±0.46	NS	NS	NS

Legend

Supplement Table S1. Lung and serum cytokine concentrations were measured by a multiplex ELISA at 0 hours (3 days after influenza influenza), 4 hours and 24 hours after MRSA challenge. Numbers: cytokine concentration Mean ± SEM (pg/mL). Concentrations of each cytokine between time-points are compared by unpaired *Student's t*-test on logarithmic data. P values < 0.05 are considered significant and reported.
