# Supplementary material for: Effect of Linezolid on Clinical Severity and Pulmonary Cytokines in a Murine Model of Influenza A and Staphylococcus aureus Coinfection
Source: PLoS One. 2013 Mar 5;8(3):e57483. doi: 10.1371/journal.pone.0057483 (PMC3589409; doi:10.1371/journal.pone.0057483)
Supplement: Table S2 — Lung and serum cytokine concentrations were measured by a multiplex ELISA at 0 hours (3 days after influenza influenza), 4 hours and 24 hours after MRSA infection. Numbers: cytokine concentration Mean ± SEM (pg/mL). Concentrations of each cytokine in the lungs and serum are compared by unpaired Student’s t-test on logarithmic data. P values <0.05 are considered significant and reported. (PDF) [file pone.0057483.s002.pdf]

Supplement Table S2. Comparison of cytokines in lungs vs serum of mice with influenza and MRSA coinfection

Cytokine (pg/mL)	0h			4h			24h		
	Lung	Serum	P	Lung	Serum	P	Lung	Serum	P
IFN-γ	33.06 ±10.85	5.42 ±0.76	0.0062	31.55 ±5.84	2.53 ±0.34	<0.0001	692.65 ±269.60	83.16 ±24.84	0.034
IL-1β	56.60 ±19.62	6.34 ±2.95	0.0244	1975.53 ±221.42	3.13 ±1.62	<0.0001	837.89 ±202.01	1.35 ±0.13	<0.0001
IL-10	15.55 ±4.65	36.69 ±7.51	NS	210.12 ±32.01	91.42 ±18.18	0.0375	27.41 ±6.45	49.28 ±3.37	0.0434
IL-12	17.00 ±3.29	95.81 ±40.49	0.0455	206.53 ±49.07	50.31 ±21.35	0.0057	46.16 ±15.55	41.94 ±5.61	NS
IL-6	186.04 ±83.35	37.59 ±14.33	NS	11767.72 ±3218.77	1735.74 ±614.42	0.0017	432.85 ±94.80	64.15 ±9.26	0.0003
mKC	116.07 ±40.42	64.08 ±11.89	NS	6594.50 ±1189.87	5058.35 ±1793.54	NS	286.09 ±45.40	210.73 ±25.40	NS
TNFα	11.37 ±4.21	2.26 ±0.75	0.0369	8388.89 ±979.42	8.97 ±3.28	<0.0001	1010.11 ±302.49	2.51 ±0.46	<0.0001

Legend

Supplement Table S2. Lung and serum cytokine concentrations were measured by a multiplex ELISA at 0 hours (3 days after influenza influenza), 4 hours and 24 hours after MRSA infection. Numbers: cytokine concentration Mean ± SEM (pg/mL). Concentrations of each cytokine in the lungs and serum are compared by unpaired *Student's t*-test on logarithmic data. P values < 0.05 are considered significant and reported.
